# Olmesartan Attenuates Tacrolimus-Induced Biochemical and Ultrastructural Changes in Rat Kidney Tissue

**DOI:** 10.1155/2014/607246

**Published:** 2014-05-28

**Authors:** Naif O. Al-Harbi, Faisal Imam, Mohammed M. Al-Harbi, Muzaffar Iqbal, Ahmed Nadeem, Mohammed M. Sayed-Ahmed, Ali D. Alabidy, Ali F. Almukhallafi

**Affiliations:** ^1^Department of Pharmacology and Toxicology, College of Pharmacy, King Saud University, P.O. Box 2455, Riyadh 11451, Saudi Arabia; ^2^Department of Pharmaceutical Chemistry, College of Pharmacy, King Saud University, Riyadh 11451, Saudi Arabia; ^3^El-Ghad International College for Health Science, Riyadh 11451, Saudi Arabia; ^4^Al-Haya Medical Co., Riyadh 11411, Saudi Arabia

## Abstract

Tacrolimus, a calcineurin inhibitor, is clinically used as an immunosuppressive agent in organ transplantation, but its use is limited due to its marked nephrotoxicity. The present study investigated the effect of olmesartan (angiotensin receptor blocker) on tacrolimus-induced nephrotoxicity in rats. A total of 24 rats were divided into four groups, which included control, tacrolimus, tacrolimus + olmesartan, and olmesartan groups. Tacrolimus-induced nephrotoxicity was assessed biochemically and histopathologically. Tacrolimus significantly increased BUN and creatinine level. Treatment with olmesartan reversed tacrolimus-induced changes in the biochemical markers (BUN and creatinine) of nephrotoxicity. Tacrolimus significantly decreased GSH level and catalase activity while increasing MDA level. Olmesartan also attenuated the effects of tacrolimus on MDA, GSH, and catalase. In tacrolimus group histological examination showed marked changes in renal tubule, mitochondria, and podocyte processes. Histopathological and ultrastructural studies showed that treatment with olmesartan prevented tacrolimus-induced renal damage. These results suggest that olmesartan has protective effects on tacrolimus-induced nephrotoxicity, implying that RAS might be playing role in tacrolimus-induced nephrotoxicity.

## 1. Introduction

Tacrolimus (TAC) is Food and Drug Administration approved immunosuppressant used clinically to reduce the rejection rate in organ transplantation. Prolonged treatment with TAC results in several adverse effects, with the most significant being nephrotoxicity [1, 2]. It has been shown previously that TAC significantly increases blood urea nitrogen (BUN) and serum creatinine level while decreasing endogenous creatinine clearance which is inversely proportional to plasma renin activity (PRA) [3, 4]. Similarly renin mRNA levels are also increased in the renal cortex in TAC-treated rats [5]. Long-term use of TAC produces histological changes which include arteriolar hyalinosis, tubular atrophy, interstitial fibrosis, thickening and fibrosis of Bowman's capsule, and focal or global glomerular sclerosis [6].

The mechanism of TAC-induced nephrotoxicity is still not well understood. Several factors may contribute to the underlying mechanisms of nephrotoxicity, which include increased production of vasoconstriction factors, such as endothelin or thromboxane, and a decrease in vasodilation factors like prostacyclin, prostaglandin E2, and nitric oxide. TAC also has the ability to produce reactive oxygen species (ROS) via activation of NADPH oxidase pathway and cause disturbance in antioxidant defense which may be responsible for nephrotoxicity [7]. It has been reported that treatment with TAC leads to impairment in renin-angiotensin system (RAS) which may mediate nephrotoxicity [8–10].

Clinical as well as animal experimental studies support the fact that angiotensin II plays a key role in pathophysiology of glomerular damage [11–13]. It has been established earlier that inhibition of Ang II may be an effective way to reverse chronic renal damage [14, 15]. For example, overexpressing renin transgenic mice shows perivascular and periglomerular inflammation in kidney and these changes are reversed by treatment with AT1 receptor antagonist [16].

Olmesartan (OLM) is an orally active angiotensin II receptor (type AT1) antagonist. Since Ang II-dependent cellular responses are mediated through ROS [17, 18], it may be possible that renoprotective effects of angiotensin receptor blockers (ARBs) are mediated through inhibition of glomerular ROS generation in chronic kidney disease [19]. Therefore we hypothesized that OLM may reduce/attenuate TAC-induced renal damage and oxidative stress through AT1 receptor antagonism. Our study focuses on the effect of OLM on TAC-induced nephrotoxicity in rats using biochemical markers of oxidative stress, renal function, and histopathological measures of cellular damage.

## 2. Materials and Methods

### 2.1. Animal Model

Male Wistar albino rats weighing 150–200 g (10–12 weeks old) were used in this study. The animals were obtained from Experimental Animal Care Center, College of Pharmacy at King Saud University. They were kept under ideal laboratory conditions (12 h light/12 h darkness cycle, 45–55% relative humidity, and temperature at 23–25°C) and maintained on standard pellet diet and water* ad libitum *during the experimental period. All experiments were carried out according to the guidelines of the Animal Care and Use Committee at King Saud University.

### 2.2. Drugs and Chemicals

TAC obtained from Sigma Aldrich, USA, and OLM obtained from Ranbaxy Research Laboratory, INDIA, were used in the study. Biochemical parameters were done using kits (Dimension, Siemens, USA). All the other chemicals used were of analytical grade.

### 2.3. Experimental Protocol

Rats were randomly divided into four groups: group 1, control group received normal saline for 14 days. Group 2, toxic group received TAC (2 mg/kg, intraperitoneally [i.p.]) for 14 days [20]. Group 3, treatment group first received TAC (2 mg/kg, i.p.) for 14 days with the same schedule as group 2 and also OLM (2 mg/kg, dissolved in distilled water and administered p.o.) for 28 days. Group 4, drug* per se* group received OLM (2 mg/kg, dissolved in distilled water and administered p.o.) for 28 days.

Rats were sacrificed by decapitation under ether anesthesia, as per the protocol. Blood sample was collected and the serum was separated by centrifugation at 3000 g for 10 min and frozen at −20°C for estimation of renal function parameters.

The kidneys were isolated and washed in ice-cold physiological saline and were used for assessment of oxidative stress, histopathology, and ultrastructural changes.

### 2.4. Biochemical Estimation

Biochemical estimations were done by autoanalyzer (Dimension RXL MAX, Siemens, USA).

### 2.5. Determination of Lipid Peroxides, Measured as Malondialdehyde (MDA)

MDA level, a product of membrane lipids peroxidation, was estimated by reacting it with thiobarbituric acid (TBA), by the method of Ohkawa et al., [21] using the standard calibration curve prepared from tetraethoxypropane. MDA was expressed as nmoles of MDA per milligram of protein. Protein was estimated by the method of Lowry et al. [22].

### 2.6. Determination of Reduced Glutathione (GSH)

GSH content was estimated by the method of Sedlack [23]. The absorbance of reaction mixture was read within 5 min of addition of 5,5′-dithiobis(2-nitrobenzoic acid) at 412 nm using UV spectrophotometer, against a reagent blank.

### 2.7. Determination of Catalase (CAT)

Kidney tissue was homogenized and PMS was used to assay CAT activity. CAT activity was estimated using the method of Clairborne [24]. The reaction mixture consisted of 1.95 mL of phosphate buffer (0.1 M, pH 7.4), 1.0 mL of hydrogen peroxide (0.019 M), and 0.05 mL of PMS in a final volume of 3 mL. Changes in absorbance were recorded at 240 nm every minute for 5 minutes. The enzyme activity was calculated as nmoles of H_2_O_2_ consumed/min/mg protein.

### 2.8. Histopathological Studies

Kidneys were harvested from the rats and fixed in 10% buffer formosaline. Paraffin sections of thickness of 3-4 *μ*m were prepared and stained with hematoxylin and eosin (H&E) for histopathological examination under light microscopy.

### 2.9. Ultrastructural Studies

Immediately after removal of kidney from the dissected rats, tissues were sliced into small size (1 mm³) and fixed in 3% buffered glutaraldehyde. Tissue specimens were then postfixed in 1% osmium tetroxide (OsO_4_) for 90 min. Dehydration of the fixed tissue was performed using ascending grades of ethanol followed by transfer of tissue to epoxy resin via propylene oxide. After impregnation with the pure resin (SPI resin), tissue specimens were embedded in the same resin mixture [25]. Ultrathin sections of silver shades (60–70 nm) were cut using an ultramicrotome (Leica, UCT, Tokyo, Japan) with a diamond knife; sections were then placed on copper grids and stained with uranyl acetate (20 min) and lead citrate (5 min). Stained sections were observed under transmission electron microscopy (JEOL JEM-1011, Tokyo, Japan) operating at 80 kV [25, 26].

### 2.10. Statistical Analysis

All results are expressed as mean + SEM. Data of groups were compared with the analysis of variance (ANOVA), followed by the Tukey-Kramer multiple comparison tests to identify significance among groups. Values were considered statistically significant when *P* < 0.05. Statistical analysis was carried out using Graph pad prism 3.0.

## 3. Results

### 3.1. Effect of OLM on TAC-Induced Changes in Parameters of Renal Function in Serum

**Figure 1:**
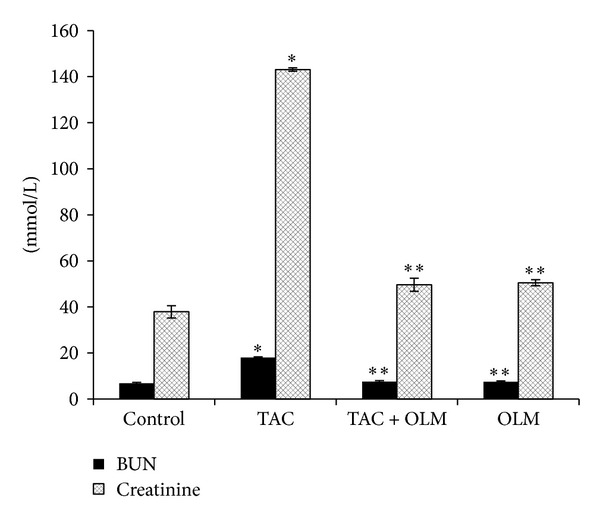
Effect of olmesartan on tacrolimus-induced changes in BUN and creatinine levels in serum of different experimental groups. The data are expressed as mean ± SEM (*n* = 6). *P < 0.05 versus control group; **P < 0.05 versus toxic group. ANOVA followed by the Tukey-Kramer multiple comparison tests.

**Figure 2:**
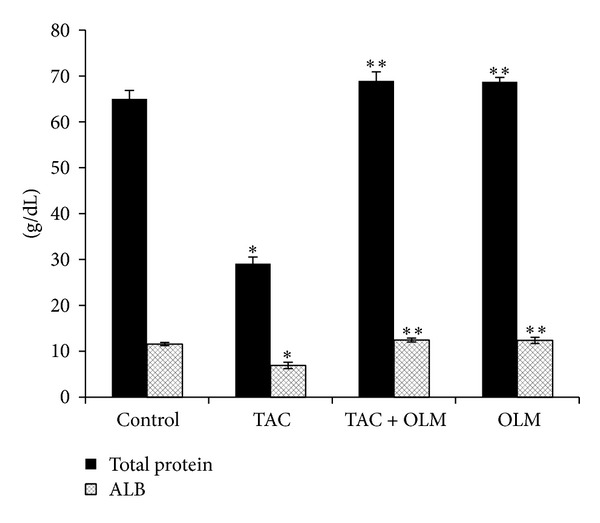
Effect of olmesartan on tacrolimus-induced changes in total protein and albumin levels in serum of different experimental groups. The data are expressed as mean ± SEM (*n* = 6). *P < 0.05 versus control group; **P < 0.05 versus toxic group. ANOVA followed by Tukey-Kramer multiple comparison tests.

Serum creatinine and BUN are biomarkers for kidney function. Treatment of rats with TAC caused renal damage as evidenced by a significant (*P* < 0.05) increase in serum creatinine and BUN levels compared to control group. Treatment with OLM significantly (*P* < 0.05) decreased both serum creatinine and BUN levels caused by TAC (Figure 1). Treatment of rats with TAC also caused significant decrease in total protein and albumin levels in serum (*P* < 0.05) compared to control group, which was reversed by OLM treatment (Figure 2). However, OLM* per se* group did not have any significant changes in any of the above parameters compared to control group (Figures 1 and 2).

### 3.2. Effects of OLM on TAC-Induced Changes in Parameters of Oxidative Stress in Kidney

**Figure 3:**
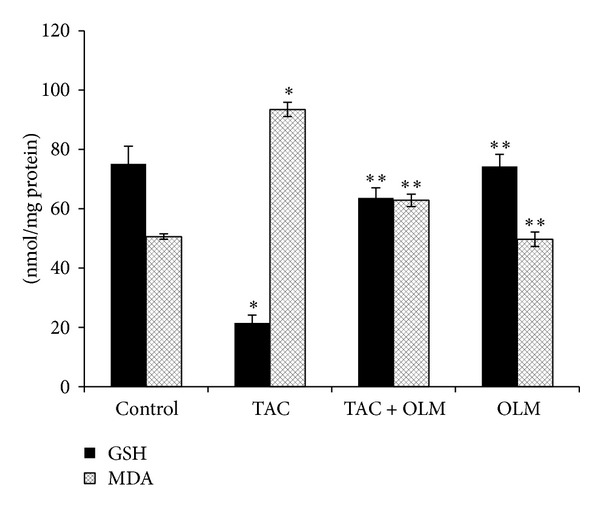
Effect of olmesartan on tacrolimus-induced changes in glutathione and lipid peroxidation in kidney of different experimental groups. The data are expressed as mean ± SEM (*n* = 6). *P < 0.05 versus control group; **P < 0.05 versus toxic group. ANOVA followed by the Tukey-Kramer multiple comparison tests.

**Figure 4:**
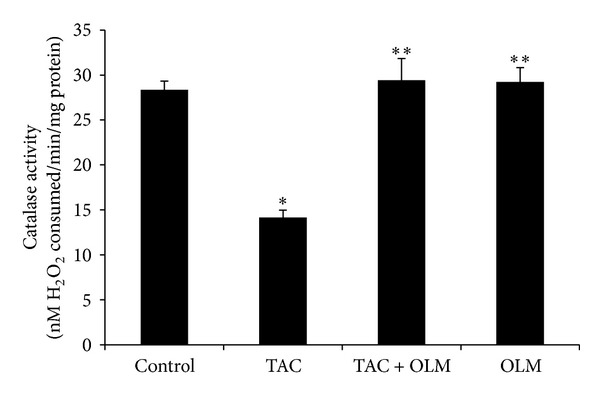
Effect of olmesartan on tacrolimus-induced changes in catalase activity in kidney of different experimental groups. The data are expressed as mean ± SEM (*n* = 6). *P < 0.05 versus control group; **P < 0.05 versus toxic group. ANOVA followed by the Tukey-Kramer multiple comparison tests.

Two-week treatments of rats with TAC resulted in a significant (*P* < 0.05) increase in kidney MDA contents compared to the control group. Treatment with OLM showed a significant (*P* < 0.05) decrease in TAC-induced kidney MDA level (Figure 3). Consequently, a significant (*P* < 0.05) decrease in kidney GSH level (Figure 3) and catalase activity (Figure 4) was found in TAC-treated group compared to control group. Treatment with OLM showed a significant improvement in TAC-induced kidney GSH level (*P* < 0.05) and catalase activity (*P* < 0.05). OLM* per se* group did not have significant changes compared to control group.

### 3.3. Effects of OLM on TAC-Induced Histopathological Changes in Kidney

**Figure 5:**
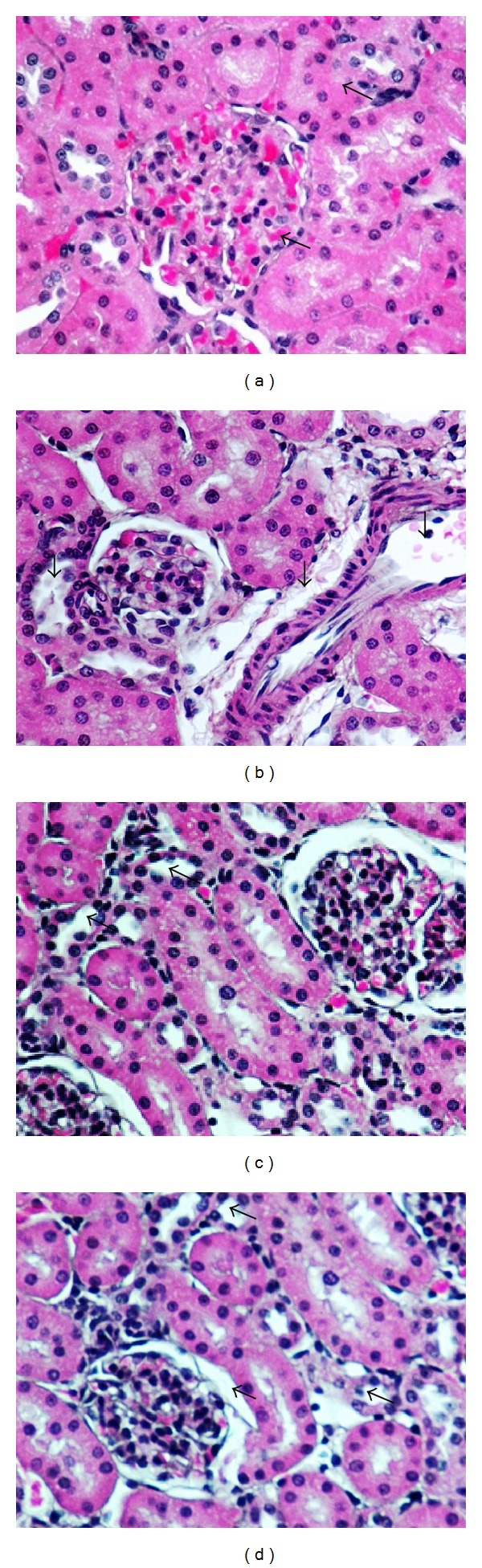
Effect of olmesartan on tacrolimus-induced changes histopathology in kidney of different experimental groups. (a) Control group; (b) toxic group; (c) treatment group; and (d) drug* per se* group (*n* = 6 per group). Magnification at 40x.

Renal tissues showed normal morphological structures in control group (Figure 5(a)). However treatment with TAC showed significant hypertrophy of epithelial cells and renal tubules epithelia degeneration. Renal glomeruli and epithelial cells enlargement in the cortical part of the kidney were also observed. In few renal tubules, single epithelial cells desquamated from the lumen were also noted (Figure 5(b)). Four-week treatment with OLM ameliorated TAC-induced damage in renal tissue compared to that observed in normal tissue (Figure 5(c)). No morphological changes were observed in OLM* per se* group (Figure 5(d)).

### 3.4. Effects of OLM on TAC-Induced Parameters of Ultrastructural Changes in Kidney

**Figure 6:**
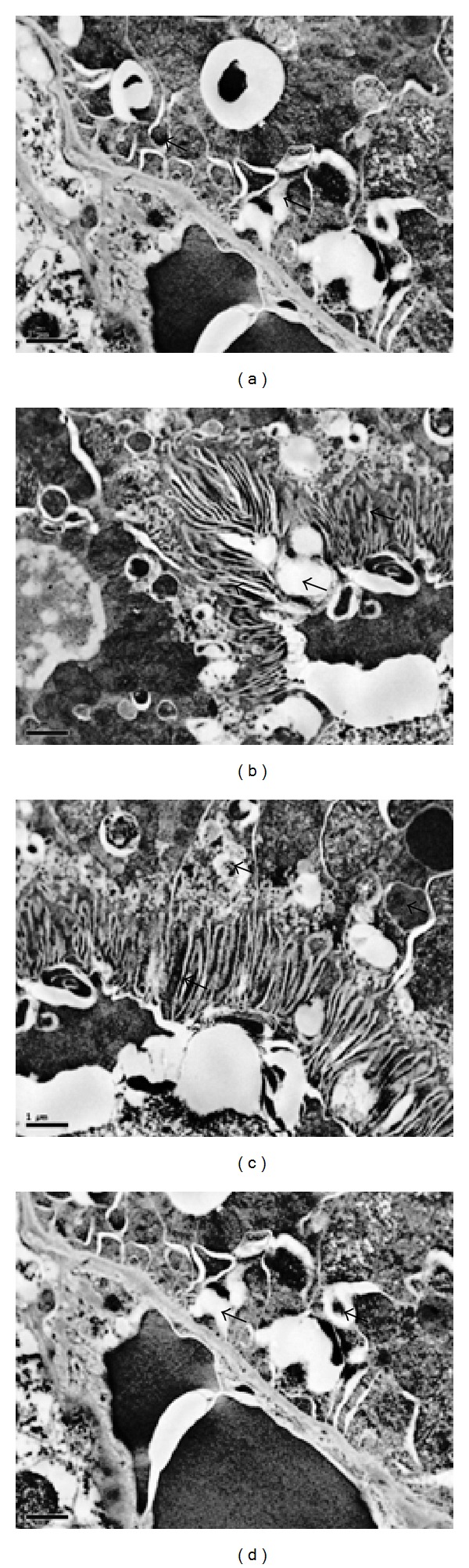
Effect of olmesartan on tacrolimus-induced ultrastructural changes in kidney of different experimental groups. (a) Control group; (b) toxic group; (c) treatment group, and (d) drug* per se* group (*n* = 6 per group). Podocyte foot process and mitochondrial integrity were assessed using transmission electron microscopy (magnification = ×10000).

Ultrastructural changes in cellular structure were further visualized by transmission electron microscopy. At ultrastructural level, normal structures of kidney were seen in control group (Figure 6(a)). By contrast, nephropathic changes were observed in the corticomedullary region of toxic group characterized by glomerular damage, including dilatation of glomerular blood vessels, exfoliation and shedding of proximal tubular cells into the tubular lumen, thickened basement membrane, and interstitial inflammation (Figure 6(b)). The dilatation of capillaries filled with erythrocytes was the most advanced change all over the kidney. Cellular damage was accompanied by “scarring” of some glomeruli (focal glomerular sclerosis) and tubules (tubular interstitial fibrosis), as well as by tubular regeneration (Figure 6(b)). We also noticed degenerated mitochondria, number of multivesicle bodies, glomerular epithelial injury, and cell debris. Under normal conditions, tubular regeneration serves to restore the loss of damaged cells by a transient increase of cell proliferation. However, in OLM-treated rats, kidneys demonstrated massive and sustained regenerative renal cell proliferation, resulting in simple tubular hyperplasia, indicated by increased number of cells and multilayered tubules. OLM treatment was associated with normalization of cellular structure and reversal of the TAC-induced renal cell damage (Figure 6(c)). Normal cellular structures were observed in OLM* per se* group (Figure 6(d)).

## 4. Discussion

The present study aimed to evaluate the effect of OLM on TAC-induced nephrotoxicity in rats using biochemical markers of oxidative stress, renal function, and histopathological measures of damage at cellular levels. The protective effects of angiotensin receptor blockers on nephrotoxicity were reported in various studies using other animal models [16, 27–30].

Previous reports have shown that TAC-induced nephropathy is manifested by elevation in the serum levels of creatinine and BUN [31–34]. In the present study, TAC-treated rats showed elevated levels of serum BUN and creatinine. However, treatment with OLM caused a significant reduction in BUN and creatinine levels which may be due to reduction in glomerular filtration damage induced by TAC. In previous study, it has been shown that concentration of creatinine and BUN depends on the glomerular filtration rate (GFR). Renal dysfunction reduces glomerular filtration of creatinine and BUN, and thus creatinine and BUN rise. If the serum creatinine and BUN levels double, the GFR is considered to have been halved [34, 35]. These results are in agreement with an earlier report [36].

Hypoalbuminemia is a characteristic feature of the nephrotic syndrome [37]. In this study serum albumin and total protein levels were found to be low in TAC-treated rats similar to previous studies [38–40]. Treatment with OLM reversed TAC-induced hypoalbuminemia and total protein levels. In a clinical trial it is reported that valsartan (ARBs) deceases the urinary albumin excretion rate to a greater extent than amlodipine, while BP was reduced to the same level. Therefore, it is suggested that OLM improves renal function by counteracting TAC-induced nephropathy.

In our study, we observed a significant increase in the concentration of MDA level in renal tissues of TAC-treated animals compared to control group. The elevated level of MDA may be due to enhanced production of ROS (superoxide radicals, hydrogen peroxide, and hydroxyl radicals). Ibrahim et al. [34] also reported a significant increase in kidney MDA content in rats with a cumulative dose of TAC [36]. Similar studies were also reported by other authors [41, 42]. Zhou et al. [4] reported that TAC leads to cell death via ROS production. In this study, treatment with OLM significantly decreased renal MDA level which suggests its role in combating oxidative stress generated by TAC. Angiotensin II plays an important role in induction and upregulation of ROS production in renal injury. Therefore OLM may have decreased TAC-induced renal damage by reduction in ROS/oxidative stress.

Decreased GSH levels and CAT activity in renal tissue may be due to excess production of ROS/lipid peroxides. In the present study, treatment with OLM reversed TAC-induced decrease in renal GSH content and CAT activity. Increased intracellular GSH content and CAT activity might be due to upregulation of enzymatic/nonenzymatic antioxidants or a decrease in oxidative stress in OLM-treated group. OLM has been shown to decrease oxidative stress in earlier studies [28, 43]. However these studies did not investigate the nephroprotective effects of OLM on TAC.

In histopathological examination, renal tubules epithelia degeneration with infiltration of mononuclear cells and dilation of glomeruli as well as hyperaemia of medullary and cortical parts was seen in toxic group. The dilatation of capillaries filled with erythrocytes was the most advanced change all over the kidney. Further, the present study showed tubular vacuolization in toxic group. Examination of renal cellular ultrastructure showed round, oval mitochondria within the tubular epithelium in TAC-treated rats. Further ultrastructural analysis shows disturbances in podocytes foot process, that is, podocyte foot injury and podocyte foot process effacement. Similar histopathological and ultrastructural changes associated with TAC-related nephrotoxicity, including tubular vacuolization, arteriolar hyalinosis, interstitial fibrosis, and juxtaglomerular hyperplasia, have been reported earlier [7, 36, 44–46]. TAC-induced histopathological and ultrastructural changes were reversed by treatment with OLM. There was restoration of podocyte architecture along with epithelial integrity after treatment with OLM. Tubular vacuolization induced by TAC was also attenuated by treatment with OLM. Earlier studies have shown that RAS blockade leads to preservation of podocytes architecture, mitochondrial function, and epithelial integrity [27, 28].

Treatment with OLM ameliorated TAC-induced nephrotoxicity implying that angiotensin receptor antagonist leads to improvement in renal function. The protective effect of OLM was accompanied by a significant attenuation of oxidative stress in kidney and improvement in renal function as well as restoration of renal structures. Thus, the current study suggests that RAS inhibition may be beneficial in case of renal damage caused by chronic use of immunosuppressant.
